# Factors influencing stress and resilience among Egyptian medical students: a multi-centric cross-sectional study

**DOI:** 10.1186/s12888-024-05820-1

**Published:** 2024-05-23

**Authors:** Hazem E. Mohammed, Zeyad Bady, Zeinab G. Abdelhamid, Bashaer Elawfi, Hadeer Elsaeed AboElfarh, Toka Elboraay, Doaa Mazen Abdel-Salam

**Affiliations:** 1 Faculty of Medicine, Assiut university, Assiut, Egypt; 2 Department of Public Health and Community Medicine, Faculty of Medicine, Assiut University, Assiut, Egypt; 3 Faculty of Medicine, Sana’a University, Sana’a, Yemen; 4 Neurology Department, Faculty of Medicine, Mansoura University, Mansoura, Egypt; 5 Faculty of Medicine, Zagazig University, Elsharqia, Egypt; 6 Medical research group of Egypt (MRGE), Cairo, Egypt

**Keywords:** Stress, Resilience, Kessler psychological distress scale, Brief resilience scale, Well-being, University students, Medical students

## Abstract

**Background:**

Psychological stress is a common psychological comorbidity among medical students and worsens their quality of life. Psychological resilience is thought to have a protective role against stress. However, evidence regarding the prevalence of stress and resilience alongside their associated factors is scarce, especially in the Middle East. This is the first multicenter, cross-sectional study to investigate resilience and stress among Egyptian medical students.

**Methods:**

The current cross-sectional study was conducted on 2465 university students in seven public universities in Egypt. The universities were selected using the simple randomization method. The data was collected using a self-administered questionnaire consisting of four parts: demographic data, socioeconomic tool represented in the Family Affluence Scale (FAS), the Kessler Psychological Distress Scale (K10), and the Brief Resilience Scale (BRS). Data was analyzed in SPSS version 26 software.

**Results:**

The majority of the students were stressed (86.5%), most of whom had severe stress (48.9%). Most of the students had low resilience (49.9%), while only 3.2% had high resilience. In the logistic regression analysis, being a female, living alone, spending long hours on social media, and thinking of suicide or leaving medicine were associated with being stressed and having low resilience. Medical students with low resilience were significantly more liable to stress [Adjusted odds ratio (AOR) = 3.667, confidence interval (CI): 2.709–4.965, *P* = 0.000], and vice versa [AOR = 3.709, CI: 2.746–5.009, *P* = 0.000]. Interestingly, high socioeconomic status showed a significant association with high resilience (*P* = 0.004); nonetheless, it was not associated with stress (*P* = 0.993). Academic grades were not associated with both the level of stress and resilience. Aging, being in clinical or academic stages, smoking, having a chronic disease, and being financially-supported are neither associated with stress nor resilience.

**Conclusions:**

The present study revealed that Egyptian medical students had low resilience and high stress, with a significant relationship between both of them. Further investigations via longitudinal study design to understand the resilience-stress relationship are recommended. Developing and implementing resilience-improving strategies in medical schools is highly recommended to decrease the prevalence of stress and its subsequent burdens.

## Introduction

Up to a third of medical students experience behavioral and psychological symptoms [1]. One of the most prominent problems is psychological stress, with an estimated prevalence of 34% [2]. Psychological stress is how our mind and emotions react when we are dealing with tough situations [3]. Stress can be a consequence of several endogenous and exogenous factors (e.g., sex, smoking, beliefs, etc.) [4]. Notably, medical students have unique stress-causing factors. These factors could be grouped into academic, socio-demographic, and psychosocial stressors. Evidently, academic stressors include having trouble understanding a new curriculum, poor educational processes, irregular schedules, frequent tests, academic stage, and anxieties about succeeding academically [5–7]. Alongside academic stressors, sociodemographic factors like gender (males versus females), smoking, substance misuse, marital status, employment during the study, parents’ educational attainment, cultural background, and family socioeconomic standing. Interestingly, cultural background might also influence the perception of stress among students [8]. In addition to the aforementioned factors, psychosocial factors like homesickness, financial hardship, and fear of failing in one’s medical career lead to inevitable and detrimental effects [9, 10].

High levels of stress in medical school have well-documented and serious effects, including impaired focus, anxiety and depression, unstable relationships, and even suicide [11, 12]. According to Seo et al., stressed medical students were almost four times more likely to commit suicide compared to non-stressed medical students [12]. However, medical students’ psychological resilience has been found to play a protective role against stress [13]. Resilience is the ability to deal with tough times and bounce back while maintaining mental and physical well-being [14]. Previous studies have shown a negative relationship between stress and resilience [15, 16]. When resilient physicians are stressed, they not only bounce back fast from stressors but also become stronger as a result [17]. Besides, resilient physicians are better time managers and have been shown to have a better life-work balance [18]. Moreover, based on the transactional stress theory, resilience and other personal traits regulate the relationship between stressors and stress responses [19]. Curiously, like stress, resilience is influenced by cultural factors. For example, a cross-sectional study among undergraduate medical students in India found that a quarter of the participants had low resilience [20]. On the other hand, a study conducted among students in Germany revealed that students have a moderate level of resilience and are not inherently prone to burnout [21]. This might raise concern for the effect of social and cultural factors on resilience levels and stress perception. Therefore, investigating stress and resilience and their predictors among the uncharted Egyptian medical students was a key driver for this study.

Investigation of stress and its correlates is scarce in the MENA region and Egypt, specifically. Despite a global prevalence of 34%, stress was prevalent in 59.9% of Assiut University students in Upper Egypt [22]. Furthermore, Ebrahim et al. reported a stress prevalence of 93% at Helwan University in the Delta region [23]. This reflects the huge, yet variable, prevalence of stress among Egyptian medical students in different geographical regions. Alongside the lack of studies and class 1 evidence about stress predictors in Egypt, we aimed to investigate stress and its predictors among multiple Egyptian medical schools.

This is the first multicentric cross-sectional study, consisting of a relatively large number of participants, to investigate resilience and stress among Egyptian medical students. To the best of the authors’ knowledge, this is the first cross-sectional study investigating resilience among medical students. The study aimed to reveal an inverse relationship between stress and resilience in the context of the socioeconomic and sociodemographic characteristics of Egyptian medical students. Moreover, the study investigated the role of some of the aforementioned academic and non-academic challenges in predicting stress and resilience. Besides, given the recent economic challenges in Egypt, investigating the role of socioeconomic status in predicting stress levels and resilience among medical students might be interesting to readers. Finally, this study highlighted some recommendations to alleviate stress among medical students in the context of the predictors of resilience.

## Methods

### Design

This study employed a descriptive, observational, multi-centric cross-sectional design, utilizing a self-administered questionnaire. The questionnaire was administered to medical students from seven different faculties of medicine at seven universities in Egypt, including Assiut University, Menia University, South Valley University, Kafr El-Sheikh University, Alexandria University, Mansoura University, and Zagazig University. The universities are located in various geographical areas across Egypt, where they were chosen based on a simple randomization process. The data collection occurred from July to October 2023.

### Participants

Medical students had to meet the inclusion criteria: (1) students in their academic years (first, second, or third years of medical school), who completed at least one semester; or students in their clinical years, who took at least one clinical rotation; and (2) the students had to be able to give informed consent. The minimum sample size was calculated using OpenEpi [24]. In a previous study conducted in Albaha, Saudi Arabia, in 2022 by Atta et al. using a similar tool (the Kessler psychological distress scale), the prevalence of stress was reported at 85.5% [25]. We aimed to detect a similar effect with an 85.5% prevalence, 5% absolute deviation, 95% confidence level, and a design effect of 1. A total of 44,644 students from the seven universities were involved in the sample size calculation, which yielded a minimum total sample size of 2465 students.

### Data collection

After calculation of the required sample size, a convenience sampling was adopted in the present study. The participants completed the questionnaire using Google Forms. The questionnaire was distributed to the students through official groups via the Telegram app and other social media platforms. The students answered anonymously, on their own time, and were not compensated to participate in the study. The questionnaire began with consent, and in order to proceed, students must fulfill the criteria beforehand. The survey was entirely in English and took, on average, 10:15 min to complete.

### Data collection tool

This self-administered questionnaire consisted of four parts: demographic data, socioeconomic tool represented in the Family Affluence Scale (FAS) [26, 27], the Kessler Psychological Distress Scale (K10) [28, 29], and the Brief Resilience Scale (BRS) [30]. The details of each part were as follows:

#### Demographic data

This section of the questionnaire aimed to collect data related to students’ demographics, including age, gender, academic level, place of residence, living conditions, academic grade in the last semester, smoking, financial support, presence of chronic illness, daily studying hours, daily hours spent on social media, and daily sleeping hours.

#### Family affluence scale (FAS)

FAS is a socioeconomic tool designed to assess the material wealth of a family, providing insights into a young person’s socio-economic background. In the beginning, the FAS first version (FAS I) comprised three items: the number of cars, the number of vacations, and having your bedroom [31]. However, it underwent many modifications to increase its validity across countries. Eventually, the most recent six-item version of the FAS was created using psychometric validation of 16 possible indicators [27]. The scale consists of six questions with “No” or “Yes” response, and each is given a score of zero or one, respectively. Then, a total score would be made after the summation of all the scores, and higher scores indicate a higher socioeconomic status. The scale showed a Cronbach’s alpha of 0.516 [32].

#### Kessler psychological distress scale (K10)

This scale quantifies overall psychological stress. The survey comprises ten positive statements and employs a 5-point Likert scale to record participants’ responses, ranging from “none of the time” (1) to “all of the time” (5). A total score is obtained based on all the responses. The scores of the ten items are then summed, yielding a minimum score of 10 and a maximum score of 50 [28, 29]. The 2001 Victorian Population Health Survey proposed a cut-off for the scale in which scores of 10:19, 20:24, 25:29, and 30:50 refer to the likelihood of being well or having a mild, moderate, or severe disorder, respectively [33]. The scale showed a Cronbach’s alpha of 0.93 [28].

#### Brief resilience scale (BRS)

This scale consists of six items that measure resilience, which refers to the capacity to recover quickly from stressful conditions. The scale ranges from 1 (strongly disagree) to 5 (strongly agree). The test has undergone cross-cultural validation and demonstrated reliability in many populations. The survey is accessible in the public domain and can be used free of charge. The score is indicative of three categories: low, normal, and high resilience, with scores of 1:2.99, 3:4.30, and 4.31:5, respectively [30]. The BRS was assessed in four samples, resulting in Cronbach’s alpha values ranging from 0.80 to 0.91 [30].

### Data analysis plan

SPSS program version 26 was used to conduct the analysis [34]. The majority of variables were categorical, such as sex, age, academic grade, and financial status, and summarized as frequencies and percentages. The present study employed a binary logistic regression to detect possible factors contributing to both stress and resilience. The independent variables involved in regression were all the sociodemographic variables and FAS. Dependent variables in regression, including stress and resilience, were grouped into stressed/non-stressed and low resilience/high-normal resilience, respectively. Furthermore, a Pearson correlation was conducted to investigate the relationship between Kessler stress and BRS scores. A P value < 0.05 was considered statistically significant.

### Ethical consideration

The study protocol was done in accordance with the Helsinki Declaration and approved by the research ethical committee of Assiut University. The IRB local approval number was (04-2023-300205). All participants had to agree before filling out the questionnaire on a consent form stating they met the eligibility criteria and were willing to proceed with the questionnaire.

## Results

### Sociodemographic characteristics and FAS

All sociodemographic characteristics are summarized in Table 1. A total of 2465 responses were collected from the seven included universities. About 59% of students were in their academic stage. The gender proportions were almost equal, with 50.7% of students being female. Students less than twenty years old constituted 47.2% of the sample. The majority (73.5%) reported living with their family or friends. Besides, the majority of the students resided in the city (59.8%). The students who had financial support (86.2%) were roughly six times those who did not. Regarding time management, the students exhibited a major tendency towards increasing hours spent on social media (more than two hours = 78.5%). On the other hand, most of the students lied in the lowest-studying-hours category of six hours or less (76.6%). Approximately 57% of students attained an academic grade of > 85% in their last semester. The preponderance of students did not report a history of having any chronic diseases, with a percentage of 90.8%. Moreover, a minority of students (5.9%) were smokers. The family affluence scale (FAS) score was normally distributed, with a mean (SD) of 6 (2.2).

### Stress and resilience

**Fig. 1:**
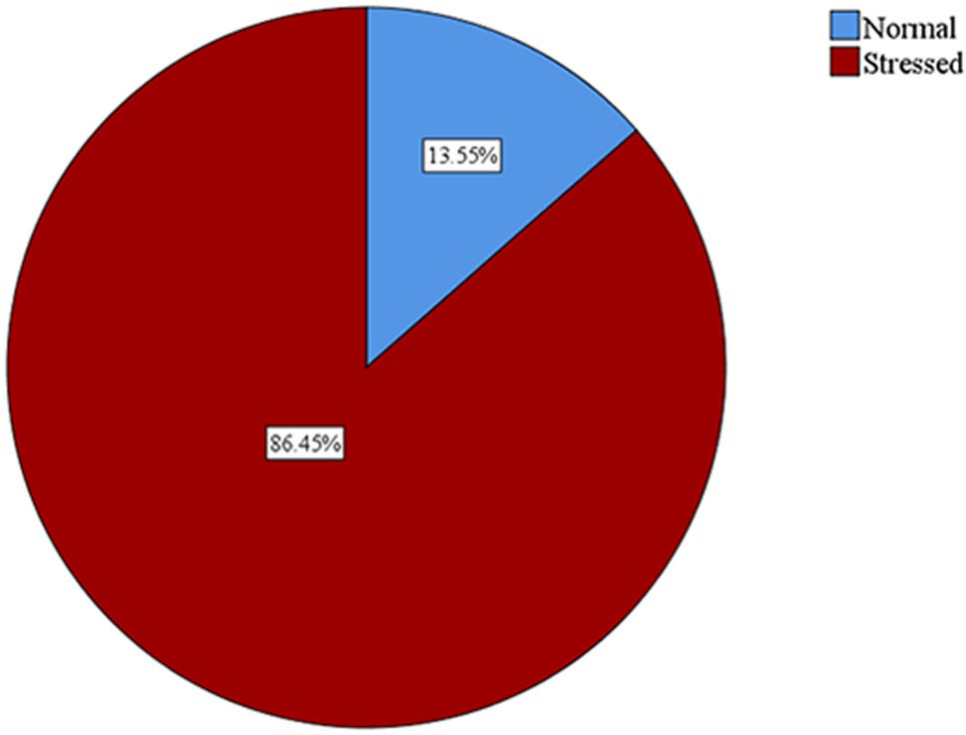
Prevalence of stress among Egyptian medical students

**Fig. 2:**
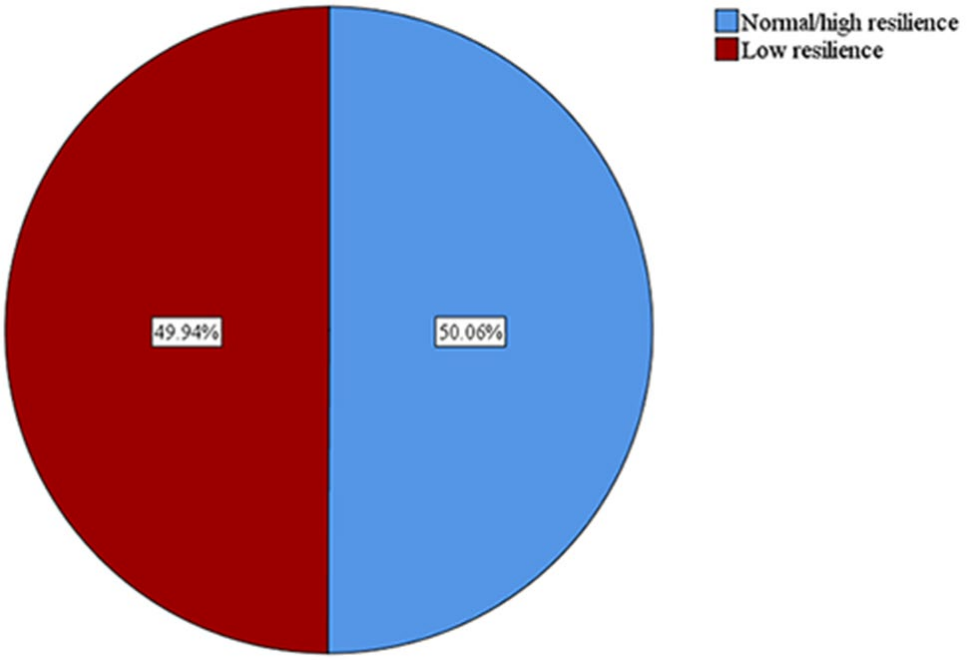
Prevalence of low resilience among Egyptian medical students

The mean (SD) for stress and resilience scores were 29.4 (8.7) and 2.9 (0.8), respectively. The stress and resilience prevalences are shown in Figs. 1 and 2. Prevalence of stress was 86.45% whereas, the prevalence of low resilience was 49.94%. Prevalences of categories of stress and resilience are represented in Table 2. Mild, moderate, and severe stress were detected among 18.1%, 19.4%, and 48.9% of the students, respectively.

**Fig. 3:**
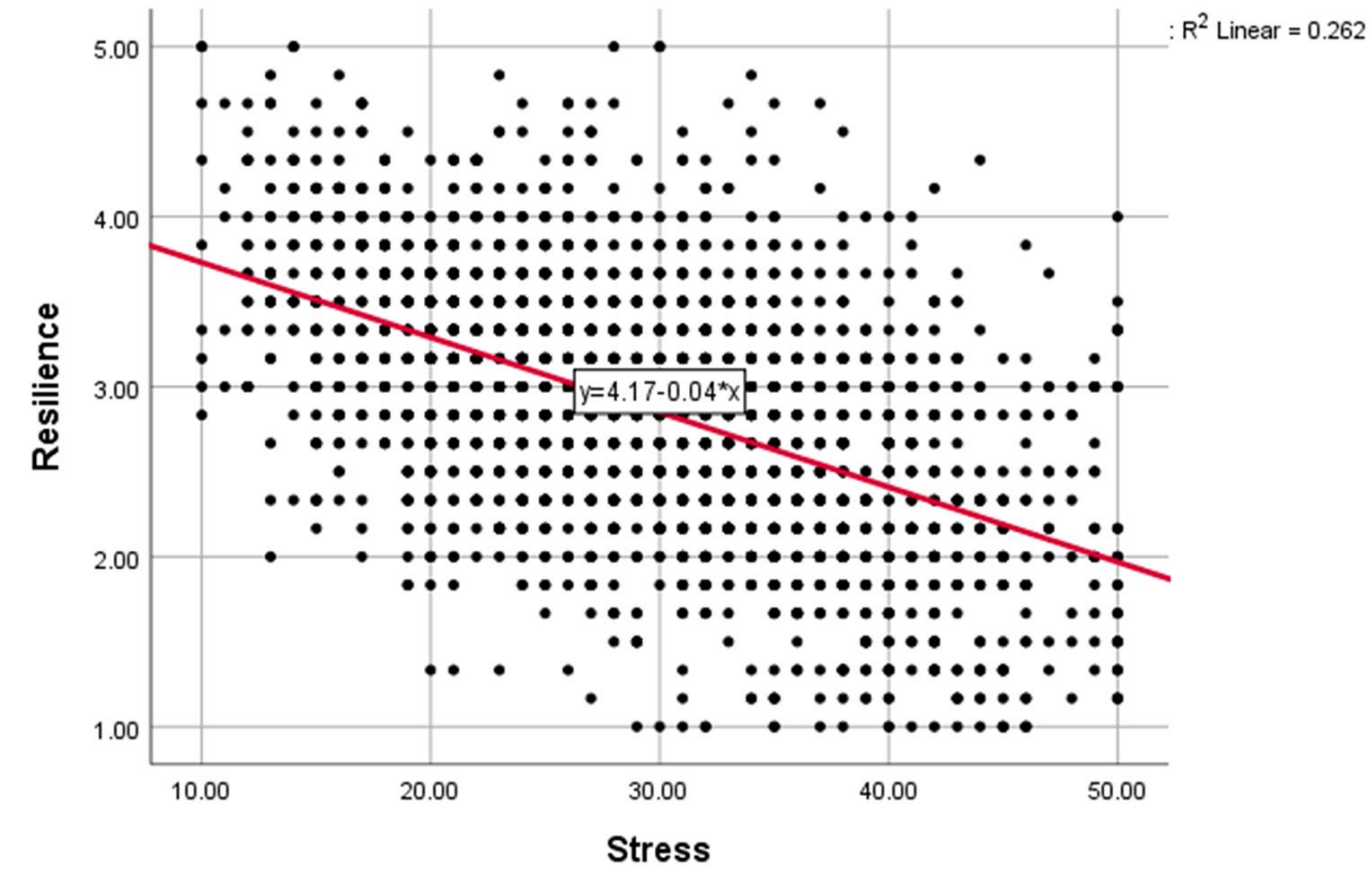
Correlation between stress and resilience among Egyptian medical students

Pearson’s correlation showed a statistically significant moderate negative correlation between stress and resilience scores, with a correlation coefficient of -0.511 and a P-value of 0.000. The scatterplot of stress and resilience is shown in Fig. 3.

**Table 1:** Sociodemographic characteristics of Egyptian medical students

Variable	Categories	Number (percentage)
Stage	Academic	1449 (58.8%)
	Clinical	1016 (41.2%)
Gender	Male	1216 (49.3%)
	Female	1249 (50.7%)
Age	< 20	1163 (47.2%)
	20:23	1075 (43.6%)
	> 23	227 (9.2%)
Residency	The city	1475 (59.8%)
	The countryside	990 (40.2%)
Living with	Alone	223 (9.0%)
	Family or friends	1813 (73.5%)
	University housing	429 (17.4%)
Academic grade in the last semester	< 75%	442 (17.9%)
	85%:75%	626 (25.4%)
	> 85%	1397 (56.7%)
Smoking	Yes	145 (5.9%)
	No	2320 (94.1%)
Social media hours daily	=<2	531 (21.5%)
	> 2	1934 (78.5%)
Studying hours daily	<=6	1887 (76.6%)
	6:9	413 (16.8%)
	> 9	165 (6.7%)
Financial support	Yes	2126 (86.2%)
	No	339 (13.8%)
Dropping out of medicine thoughts	Always	308 (12.5%)
	Sometimes	726 (29.5%)
	Rare/Never	1431 (58.1%)
Suicidal thoughts in the past month	Always	161 (6.5%)
	Sometimes	339 (13.8%)
	Rare/Never	1965 (79.7%)
Chronic disease	Yes	228 (9.2%)
	No	2237 (90.8%)
Sleeping hours	less than 6 h	450 (18.3%)
	from 6 to 8 h	1557 (63.2%)
	more than 9 h	458 (18.6%)

**Table 2:** Prevalence of stress and resilience among Egyptian medical students

Scale and categories		Number (percentage)
Stress	Well	334 (13.5%)
	Mild stress	447 (18.1%)
	Moderate stress	479 (19.4%)
	Severe stress	1205 (48.9%)
Resilience	Low resilience	1231 (49.9%)
	Normal resilience	1154 (46.8%)
	High resilience	80 (3.2%)

### Factors associated with stress and resilience

Table 3 revealed that stress among medical students was significantly associated with gender, hours of studying, social media hours, living with family or friends, dropping out of medicine thoughts, suicidal thoughts, and resilience in the logistic regression analysis. Regarding sex, females were significantly at risk of stress compared to males (Adjusted odds ratio (AOR) = 2.157, confidence interval (CI): 1.647–2.824, *P* = 0.000). Studying for 6 to 9 h and more than 9 h were significantly associated with stress (AOR = 1.824, CI: 1.255–2.652, *P* = 0.002) and (AOR = 2.235, CI: 1.202–4.153, *P* = 0.011), respectively, compared to studying for less than 6 h. Spending more than 2 h on social media was significantly associated with stress (AOR = 1.610, CI: 1.214–2.137, *P* = 0.001). Furthermore, living with family or friends was significantly less associated with stress compared to living alone (AOR = 0.536, CI: 0.298–0.966, *P* = 0.038). Having thoughts about dropping out of medicine either sometimes or always were positively associated with stress (AOR = 2.288, CI: 1.628–3.1216) and (AOR = 3.218, CI: 1.681–6.161), respectively, compared to never/rarely having them. Both were statistically significant (*P* = 0.000). Having suicidal thoughts either sometimes or always was a significant risk factors for stress (AOR = 6.685, CI: 2.691–16.607, *P* = 0.000) and (AOR = 3.290, CI: 1.010–10.716, *P* = 0.048), respectively, compared to never/rarely having them. Moreover, medical students with low resilience were significantly more liable to stress (AOR = 3.667, CI: 2.709–4.965, *P* = 0.000).

Table 4 revealed that resilience among Egyptian medical students was significantly associated with gender, hours of studying, social media hours, sleeping hours, dropping out of medicine thoughts, suicidal thoughts, stress, and FAS in the logistic regression analysis. Concerning sex, being of the female gender was positively associated with low resilience (AOR = 1.521, CI: 1.278–1.810, *P* = 0.000). Furthermore, studying for 6 to 9 h was significantly associated with higher resilience compared to studying for less than 6 h (AOR = 0.793, CI: 0.629–0.999, *P* = 0.049). Spending more than 2 h on social media was significantly associated with low resilience (AOR = 1.251, CI: 1.012–1.547, *P* = 0.038). Furthermore, sleeping for 6 to 9 h was positively associated with higher resilience compared to sleeping more than 9 h (AOR = 0.523, CI: 0.603–0.951, *P* = 0.017). Having thoughts about dropping out of medicine either sometimes or always was significantly associated with lower resilience (AOR = 1.468, CI: 1.207–1.787, *P* = 0.000) and (AOR = 1.413, CI: 1.067–1.871, *P* = 0.016), respectively, compared to never/rarely having them. Having suicidal thoughts either sometimes or always were significant risk factors for lower resilience (AOR = 2.025, CI: 1.558–2.632) and (AOR = 2.066, CI: 1.416–3.015), respectively, compared to never/rarely having them. Being stressed was strongly associated with lower resilience (AOR = 3.709, CI: 2.746–5.009, *P* = 0.000). Regarding the family affluence scale, a higher scale score indicating high socioeconomic status was significantly protective to reduced resilience (AOR = 0.943, CI: 0.905–0.982, *P* = 0.004).

**Table 3:** Factors associated with stress among Egyptian medical students

Independent Variables	AOR (95% CI)	*P*-value
**Gender:**		
Male (r)	–	–
Female	2.157 (1.647–2.824)	0.000*
**Academic grade**		
< 75% (r)	–	
85%:75%	1.050 (0.664–1.662)	0.834
> 85%	0.715 (0.477–1.073)	0.106
**Hours of studying**:		
< 6 (r)	–	–
6:9	1.824 (1.255–2.652)	0.002*
> 9	2.235 (1.202-4.153)	0.011*
**Social media hours**:		
=<2 (r)	–	–
> 2	1.610 (1.214–2.137)	0.001*
**Sleeping hours**:		
> 9 (r)	–	–
6:9	0.759 (0.527–1.093)	0.138
< 6	1.645 (0.985–2.746)	0.057
**Living with**:		
Alone (r)	–	–
Family or friends	0.536 (0.298–0.966)	0.038*
University housing	0.636 (0.330–1.225)	0.176
**Dropping out of medicine thoughts**:		
Never/Rare (r)	–	–
Sometimes	2.288 (1.628–3.216)	0.000*
Always	3.218 (1.681–6.161)	0.000*
**Suicidal thoughts**:		
Never/Rare (r)	–	
Sometimes	6.685 (2.691–16.607)	0.000*
Always	3.290 (1.010-10.716)	0.048*
**Financial support**		
Yes (r)	–	–
No	1.443 (0.934–2.229)	0.099
**Chronic diseases**:		
No (r)	–	
Yes	1.514 (0.869-2.639)	0.143
**Age**		
< 20 (r)	–	–
20:23	1.351 (0.912–2.002)	0.133
> 23	1.745 (0.955-3.188)	0.070
**Residency**		
countryside (r)	–	–
city	0.902 (0.689-1.181)	0.452
**Smoking**		
Yes (r)	–	–
No	1.082 (0.601-1.949)	0.792
**Stage**		
Clinical (r)	–	–
Academic	1.281 (0.851–1.927)	0.235
**Resilience**		
Normal/high (r)	–	–
Low resilience	3.667 (2.709–4.965)	0.000*
**Family affluence scale**	1.000 (0.940–1.064)	0.993

AOR: adjusted odds ratio; (r): reference; ^*^: statistically significant p-value less than 0.05

**Table 4:** Factors associated with low resilience among Egyptian medical students

Independent Variables	AOR (95% CI)	*P*-value
**Gender:**		
Male (r)	–	–
Female	1.521 (1.278–1.810)	0.000*
**Academic grade**		
< 75% (r)	–	–
85%:75%	1.054 (0.807–1.377)	0.698
> 85%	0.921 (0.723–1.173)	0.507
**Hours of studying**:		
< 6 (r)	–	–
6:9	0.793 (0.629-0.999)	0.049*
> 9	0.844 (0.602–1.182)	0.323
**Social media hours**:		
=<2 (r)	–	–
> 2	1.251 (1.012–1.547)	0.038*
**Sleeping hours**:		
> 9 (r)	–	–
6:9	0.523 (0.603-0.951)	0.017*
< 6	0.912 (0.688 − 1.210)	0.523
**Living with**:		
Alone (r)	–	–
Family or friends	0.778(0.571–1.060)	0.112
University housing	0.906 (0.631–1.299)	0.590
**Dropping out of medicine thoughts**:		
Never/Rare (r)	–	–
Sometimes	1.468 (1.207–1.787)	0.000*
Always	1.413 (1.067–1.871)	0.016*
**Suicidal thoughts**:		
Never/Rare (r)	–	–
Sometimes	2.025 (1.558–2.632)	0.000*
Always	2.066 (1.416–3.015)	0.000*
**Financial support**		
Yes (r)	–	–
No	0.907 (0.706-1.165)	0.443
**Chronic diseases**:		
No (r)	–	–
Yes	1.122 (0.836-1.507)	0.443
**Age**		
< 20 (r)	–	–
20:23	1.246 (0.974–1.593)	0.080
> 23	1.259 (0.858-1.849)	0.240
**Residency**		
countryside (r)	–	–
city	0.997 (0.832-1.193)	0.971
**Smoking**		
Yes (r)	–	–
No	1.366 (0.938-1.991)	0.104
**Stage**		
Clinical (r)	–	–
Academic	1.117 (0.865–1.441)	0.397
**Stress**		
Normal (r)	–	–
Stressed	3.709 (2.746–5.009)	0.000*
**Family affluence scale**	0.943 (0.905–0.982)	0.004*

AOR: adjusted odds ratio; (r): reference; ^*^: statistically significant p-value less than 0.05

## Discussion

### Stress prevalence and associated factors

Psychological health among medical students in Egypt lacks thorough investigation and research in many aspects, emphasizing the present study’s importance. The present study showed a high prevalence of stress among medical students (86.5%); the majority of them fall into the severe stress category (48.9%). Studies conducted in other regions of the Middle East were consistent with our study regarding the magnitude of stress; a study conducted in Saudi Arabia by Atta et al., using the K10 scale as our study, revealed a stress prevalence of 85.5% [25]. However, a study conducted in Peru by Valladares-Garrido et al. revealed a stress prevalence of 62.7% [35]. The slight variability in the prevalence of stress, despite the agreement on stress magnitude, is multifaceted. For example, different scales used in stress assessment may introduce different prevalence estimations. Besides, different cultures can significantly influence perceiving and coping with stress. For instance, a prior study found out Japanese undergraduates were more likely than European Canadians to have psychological and physical stress symptoms in interpersonal situations. However, there was no difference in the risk of having either type of symptom in non-interpersonal scenarios [8]. This logic could interpret some of the differences between the stress prevalence in our study and that of Valladares-Garrido et al [35]. In comparison to existing studies in the region, stress prevalence of 86.45% in this study lies notably between the reported rates by Fawzy et al. (59.9%) [22] and Ebrahim et al. (93.2%) [23]. In the present study, the prevalence appears higher than that reported by Fawzy et al. and slightly lower than that observed by Ebrahim et al. Several potential reasons could account for these discrepancies. Variations in sample demographics, such as age, gender distribution, and socio-economic status, may influence stress levels. Methodological differences, including variations in assessment tools, could contribute to divergent prevalence rates. Furthermore, the two aforementioned studies only included students from a single center, potentially limiting the generalizability of their findings.

The present study showed that being female was a significant risk factor for stress (AOR = 2.157), which is consistent with Fawzy et al. [22] and Atta et al. [25] findings. According to the American Psychological Association, women report higher stress levels than men and are more likely to experience physical and emotional symptoms of stress [36]. Many theories explain females’ dominance for both stress and depression compared to males, including gonadal hormones. Female sex hormones tend to attenuate the Hypothalamic-Pituitary-Adrenal Axis and thus minimize and delay cortisol action in the brain, resulting in less stress containment [37]. Other psychosocial factors like role stress, victimization, and sex-specific socialization have been discussed [38].

Academic grades were not significantly associated with stress in our regression analysis, which is similar to Fawzy et al.‘s findings. Nevertheless, we believe that academic performance is one of the serious factors related to stress because joining post-graduate training and residency in prestigious university programs in Egypt is only guaranteed by high grades. Besides grade, a prior study supported our results concerning study hours, in which an increasing number of study hours showed a significant association with stress development [22]. Another study showed a positive correlation between stress and the number of study hours [39]. All these findings could be attributed to decreased social activities and/or sleeping hours. Therefore, a complex sequela, including disturbances in cortisol levels, circadian rhythm, and metabolism, follows [40].

This study found students who reported living with their family or friends exhibited less liability to be stressed compared to being alone. Loneliness in the long term not only causes both acute and chronic stress, but also impairs immunity [41, 42]. Moreover, more than two hours spent on social media was a significant risk factor for stress, according to our results. Although previous studies showed an association between social media and mood fluctuations [43, 44], which is in line with ours, social media is still debatable as it is nowadays considered a paramount education and experience exchange platform.

Having suicidal thoughts was a significant risk factor for stress development in this study. This is in line with a previous finding revealed that mental distress was the most important factor associated with suicidal ideation [45]. Medical students tend to have suicidal ideation and attempts, estimated to be three times higher than that for the age-matched general population [46]. Similarly, having thoughts about leaving medicine exhibited a statistically significant association with stress. In fact, it was documented in a study in Switzerland that one in twelve physicians showed increased burnout, and every sixth one thought of leaving medicine [47]. Although Switzerland is a high-income country, unlike Egypt, an effort-reward imbalance strongly correlated with leaving thoughts. Surprisingly, the family affluence scale was statistically insignificant factor of stress in our study. This agrees with a study in Iraq that showed a statistically insignificant association between monthly income and level of stress [48].

### Resilience prevalence and associated factors

Our study showed low and high resilience prevalences of about 49.9% and 3.2%, respectively. In accordance with our results, a study conducted in Oman revealed similar prevalences of low and high resilience of 45.3% and 5.5%, respectively [49]. Basically, resilience research has primarily focused on Western-based outcomes, and there is a lack of cross-cultural validation of findings and rigorous investigations associated with resilience in non-western cultures [50]. Resilience is a dynamic and multidimensional construct that is influenced, mainly since birth, by a child’s environment and the interaction between individuals and their social ecologies. For instance, a study explored the effect of a culture- and gender-specific intervention program on the resilience of African American girls. Eventually, the results indicated that culture significantly correlated positively with resilience [51]. According to another study, strong gender and ethnic identities are directly associated with increased resilience [52, 53]. Additionally, spiritual practice, life satisfaction, and coping mechanisms play a role in resilience development. Therefore, resilience is a specific trait for each population. Since Egyptian medical students’ resilience assessment is unprecedented in any previous study, comparisons with Western cultures might not be fruitful.

Our results showed that gender exhibited a statistically significant relationship with low resilience, whereas females were more liable to low resilience. This contradicts a previous study in Oman, which showed no significant difference in the level of resilience across genders [49]. However, it has been shown that males are more resilient than females in general [54–56]. According to our results, females are 1.5 times more vulnerable to low resilience compared to males. Besides gender, our study revealed that sleeping six to nine hours is protective against low resilience, which agrees with a previous study [49].

Suicidal ideation was a significant risk factor for developing low resilience in our surveyed students. Literature has supported, on many occasions, the buffering ability of resilience against suicidal ideation and attempts, especially among those with depression or anxiety [57, 58].

Since the COVID-19 pandemic, many students have increased their screen time on social media apps, even after the pandemic wanes. A study disclosed that reduced use of digital technology in general was a significant factor in increasing resilience against suicide [59]. Regarding our results, we found that spending more than two hours on social media had an increased risk factor for low resilience, suggesting coherence with the aforementioned literature. Moreover, the family affluence scale was a significant protective variable for low resilience in our study. It is intuitive to presume that a high socioeconomic status will strengthen resilience and a sense of well-being. However, previous studies were controversial and contradictory regarding the socioeconomic effect on resilience [20, 60, 61]. This could be attributed to different financial levels among countries and even variable monthly incomes between other geographical areas in the same country.

Resilience has been reported to be higher in non-medical students compared to medical students, who had significantly higher psychological distress [62]. Our results succeeded in demonstrating that students with low resilience were significantly more likely to develop stress and vice versa. Our study, in agreement with other studies [49], showed a negative relationship between stress and resilience. However, determining whether resilience or stress is the cause of the other is unapproachable, considering our study design. Resilience, a pivotal trait, operates as a psychological stronghold against other disorders. Not only is stress correlated with resilience, as shown in our study, but depression as well. A previous study confirmed that psychological resilience was significantly negatively correlated with depression, anxiety, and somatization symptoms [63]. The interplay between resilience and psychological disorders like stress and depression is dynamic; people vulnerable to stress are more likely to develop depression and anxiety [64–67]. Consequently, stress resilience and depression resilience are highly associated, and individuals with high stress resilience are more likely to be exempted from depression [68–71].

### Strengths, limitations, and recommendations

Our study is the first to assess resilience among medical students in Egypt and investigate its associated factors like stress and other sociodemographic characteristics, including academic and non-academic stressors. Moreover, our study design is multi-centric, comprising different universities from upper and lower Egypt, in an attempt to provide generalizability and reliability to the results. We took the first initiative to investigate the effects of academic/clinical stages on students’ stress and resilience.

However, because limitations are inevitable for any study, our study also had ones. Our cross-sectional design captures data at a single point in time. Accordingly, it was impossible to establish a temporal relationship between stress, resilience, and associated factors. As stress and resilience are dynamic processes that can change over time in response to various factors, cross-sectional studies provide only a snapshot of these processes. Therefore, it’s unclear whether stress leads to lower resilience or vice versa. While questionnaires are efficient, they might lack the depth and nuance that qualitative methods, such as interviews, can provide. Accordingly, questionnaires lack the ability to follow up on ideas and clarify issues. Another drawback of the questionnaires is the potential for recall bias, where participants may have difficulty in recalling past experiences or emotions, affecting the reliability of reported stress levels and resilience factors. An additional critical bias is the social desirability bias, where participants may provide responses that they perceive as socially desirable rather than reflecting their authentic experiences or feelings, particularly when reporting on sensitive topics like stress and resilience. Furthermore, this study did not consider personality traits or baseline information about the mental status of medical students at the time of enrollment in medical schools.

Therefore, we recommend that future studies focus on: (1) conducting prospective longitudinal and cohort studies to determine the temporal sequence of the variables so they can monitor people longitudinally and determine the chronological connections among stress, resilience, and related factors. This method will empower researchers to gain deeper insights into the dynamics and interplay of these variables over time, facilitating more robust causal conclusions; (2) comparing medical to non-medical students, especially in the Middle East and Egypt; (3) mixed-methods research that could be beneficial, incorporating qualitative data collection to complement the quantitative findings, like doing interviews with students besides questionnaires. Hence, they can provide participants with the opportunity to elaborate on their responses, allowing for a more comprehensive analysis; (4) implementing diverse sampling strategies to ensure the inclusion of a wide range of participants, including those from different demographic backgrounds, socioeconomic statuses, and cultural contexts that may shape individuals’ experiences of stress and resilience. This will enhance the generalizability of findings; and (5) furthermore, our study findings propose that universities should take a proactive approach by developing and implementing comprehensive resilience-building programs tailored to the unique needs of their student populations. By developing and implementing resilience-building programs that encompass stress management workshops, counseling services, and integration of resilience-building components into the medical curriculum, universities can play a pivotal role in supporting the mental health and well-being of their students and fostering a resilient academic community. Universities can also review and adjust academic policies and practices to reduce excessive academic pressure on students. Moreover, considering mindfulness practices, fitness facilities, recreational activities, and nutrition education can help students develop resilience skills.

## Conclusion

Stress and resilience were thoroughly investigated in seven Egyptian public universities from all over Egypt. The majority of the students were stressed and had low resilience. Being a female, living alone, spending long hours on social media, and thinking of suicide or leaving medicine were strong factors associated with being stressed and having low resilience. Students with low resilience were more likely to be stressed, and vice versa. Academic grades were not associated with the level of stress. Interestingly, high socioeconomic status showed a strong relationship with high resilience; nonetheless, it was not associated with stress. Finally, we recommend further investigations regarding resilience and its associated factors. We also encourage the universities to invest time and effort in developing those factors for a better and healthier educational environment.

## Data Availability

All data generated or analyzed during this study are included in this published article.

## Abbreviations

FAS: Family Affluence Scale
K10: Kessler Psychological Distress Scale
BRS: Brief Resilience Scale
AOR: Adjusted odds ratio
CI: Confidence interval
